# Systolic MOLLI T1 mapping with heart rate depends pulse sequence sampling scheme is feasible in patients with atrial fibrillation

**DOI:** 10.1186/1532-429X-18-S1-P278

**Published:** 2016-01-27

**Authors:** Xiaohai Ma, Lei Zhao, Tianjing Zhang, Jing An, Andreas Greiser, Zhanming Fan

**Affiliations:** 1 Radiology, Beijing Anzhen Hospital, Beijing, China; 2 Siemens Ltd, Beijing, China

## Background

T1 mapping enables assessment of myocardial characteristics. As the most common type of arrhythmia, atrial fibrillation (AF) often accompany with variety of cardiac pathology, which may cause inaccurate T1 estimation due to mistriggering and inadequate magnetization recovery. We hypothesized that systolic T1-mapping with heart rate depends pulse sequence scheme may overcome this issue.

## Methods

21 patients with AF were enrolled and underwent cardiac magnetic resonance (CMR) exam at 3.0 T, 3 of these patients repeated CMR exam after electric cardioversion. A Modified Look-Locker Inversion Recovery (MOLLI) sequence was acquired before and 15 min after administration of 0.1 mmol/kg gadopentetate dimeglumine. Both of the fixed sampling scheme (5(3)3) and heart rate depends sampling scheme were performed at diastole and systole respectively. The heart rate depends pulse sequence sampling scheme was 5(n)3, n was determined by the heart rate to ensure the adequate magnetization recovery. T1 times were measured in myocardium and blood. Extracellular volume fraction (ECV) was calculated.

## Results

For 5(3)3, the average native T1 times / ECV in the basal, mid-ventricle and apex were 1277.5 + 35.7 ms / 26.9 + 2.9 %, 1269.3 + 38.3 ms / 26.6 + 2.5 %, 1268.7 + 47.4 ms / 28.1 + 3.0 % in the diastolic phase, and 1275.3 + 51.1 ms / 26.5 + 3.3 %, 1259.5 + 48.2 ms / 26.4 + 2.6 %, 1264.8 + 40.7 ms / 28.1 + 2.9 % in the systolic phase; For 5(n)3, the average native T1 times in the basal, mid-ventricle and apex were 1313.7 + 52.7 ms / 28.3 + 4.3 %, 1312.0 + 52.6 ms / 27.8 + 2.7 %, 1311.4 + 47.8 ms / 29.2 + 3.4 % in the diastolic phase, and 1313.7 + 55.5 ms / 27.1 + 3.3 %, 1305.1 + 70.7 ms / 27.2 + 3.7 %, 1304.2 + 61.8 ms / 28.1 + 3.3 % in the systolic phase; there were statistic significant differences of native T1 times and ECV between 5(3)3 and 5(n)3 both in diastolic and systolic phase, the native T1 times and ECV of 5(n)3 were larger than 5(3)3. After electric cardioversion, the 3 patients' average native T1 times / ECV in the basal, mid-ventricle and apex were 1299.7 + 36.5 ms / 24.4 + 1.6 %, 1312.4.6 + 40.1 ms / 24.1 + 0.4 %, 1325.7 + 50.8 ms / 26.3 + 0.5 % in the diastolic phase, and 1296.0 + 37.6 ms / 24.3 + 0.4 %, 1300.4 + 37.5 ms / 25.4 + 0.1 %, 1324.2 +63.6 ms / 24.9 + 0.4 % in the systolic phase, which were more similar to 5(n)3.

## Conclusions

Systolic MOLLI T1 mapping with heart rate depends pulse sequence scheme is accurate and feasible in AF and may extend clinical applicability to cardiac pathology with tachyarrhythmia.

